# A CRISPR Resource for Individual, Combinatorial, or Multiplexed Gene Knockout

**DOI:** 10.1016/j.molcel.2017.08.027

**Published:** 2017-09-21

**Authors:** Nicolas Erard, Simon R.V. Knott, Gregory J. Hannon

(Molecular Cell *67*, 348–354; July 20, 2017)

In our manuscript, we erroneously labeled sequences that were downloaded using the Dharmacon CRISPR RNA Configurator website as being selected with the Edit-R algorithm. These sequences were not selected by the Edit-R algorithm, nor were any Dharmacon products used in this manuscript. As a result, we are unable to draw conclusions regarding the efficacy of the Edit-R algorithm relative to the CRoatan algorithm. We apologize for any confusion this may have caused.

